# Supplementary material for: Neuroimaging ‘will to fight’ for sacred values: an empirical case study with supporters of an Al Qaeda associate
Source: R Soc Open Sci. 2019 Jun 12;6(6):181585. doi: 10.1098/rsos.181585 (PMC6599782; doi:10.1098/rsos.181585)
Supplement: Supplementary Information on Neuroimaging Will to Fight [file rsos181585supp1.docx]

Hamid et al., Neuroimaging “Will to Fight”

**Supporting Information**

# S1. Methods

## S1.1. Pre-selection field survey

We conducted fieldwork from early 2014 to mid-2015 with the Pakistani immigrant community in Barcelona to understand its general cultural context (including political and religious sensitivities) through participant observation, and through in-depth interviews to identify appropriate respondents for an fMRI study of extremists (in this case, supporters of militant jihad) and their willingness to make costly sacrifices for their values. For the fMRI study, we needed to: a) identify relevant values connected with jihadism (and, for comparison, other values connected to religion), b) develop appropriate measures of these values valid for this community, and c) identify those respondents who were devoted to these values (but not to the comparison values).

The first stage began with informal and semi-structured interviews with dozens of community members and leaders (imams, social workers, lawyers, community leaders and chapter heads of political parties). These in-depth interviews yielded values and issues relevant to militant jihad (e.g., expulsion of Western forces from Muslim countries), as well as issues concerning grievances related to religious belief (e.g., availability of Halal food in Spanish public schools) and other issues important to the community but not likely to be sacred. The fMRI design required at least 6 sacred values and 6 non-sacred values for comparison; and we identified 14 issues total (see section on values below).

In the second stage, a pilot questionnaire probed for sacredness regarding these 14 values. We initially used a measure of sacred values established in studies on intergoup conflict (1), wherein participants are asked if they can imagine circumstances where they would compromise the given value for a material benefit. However, our fieldwork revealed that many of our respondents were hesitant about this approach because they could not readily imagine concrete examples of how such a material benefit might look. Accordingly, we asked interviewees to come up with possible situations that would lead them to not pursue the given value anymore. Based on responses, we developed a set of specific case-based probes involving a concrete material benefit (e.g., Muslim countries legalizing gay marriage to gain foreign aid). The final measure for sacred values consisted of case-based probes for each given value (see interview instrument in Appendix). No comparable difficulties arose with the other measures (e.g., Identity Fusion). We also identified a list of sacrifices that respondents were realistically willing to make, ranging from peaceful democratic means (protesting) to violent means (joining a militant group). For each of these sacrifices, we probed for every given value to produce a score for willingness to sacrifice. At the end of the interview, we administered a radical attitudes inventory and a brief Big Five personality inventory, allowing comparison of fMRI participants with other respondents.

The third stage (mid 2015) involved face-to-face interviews with male Pakistanis in neighborhoods in and around Barcelona that have a high proportion of Pakistani immigrants (El Raval, Badalona, Barceloneta, Terrassa, etc). After obtaining oral consent, respondents were given face-to-face interviews with a field researcher and an interpreter (see section on Method for measures). Respondents had copies of the instrument, so they could see the visual measures and ensure their responses were recorded correctly. Interviews were conducted in either Urdu or Punjabi (whichever respondents were more comfortable with; there was no Punjabi version of the instrument because Punjabi is not a written language in Pakistan but all respondents were capable of reading Urdu). Each interview took 45-60 minutes to complete. Respondents were compensated for their time with a payment of €20 and were informed that, if selected for the fMRI study, they could earn an additional €100. Respondents were not informed about selection criteria for the fMRI study, as this could have affected responses. After the interviews, respondents were debriefed and encouraged to provide feedback.

### *Demographics of the pre-selection sample*

The total sample consisted of only male respondents, N = 146, average age *M* = 30.82 years (range 18 to 62). Fig S1A, B and C give distributions of marital status, education, and household income. All respondents were Muslims; most self-identified as Sunni. Fig S1D breaks down the sample’s religious make-up. The project’s focus concerned support for militant jihadism, which is a Sunni movement. Thus, Shia were excluded from analyses, resulting in an effective sample of N = 136. Fig S1E and F depict private prayer and mosque attendance frequency, respectively.

**Fig S1.** **Demographics of the pre-selection sample**. **(A)** Marital status, **(B)** education level, **(C)** income, **(D)** religious sect, **(E)** private prayer frequency and **(F)** mosque attendance frequency.

## S1. 2. Distribution of Sacredness and Sacrifices

Respondents were presented with 14 issues and probed if they considered them sacred by asking whether they would give up each for material gain. Issues were probed for sacredness were: Belief in Allah (blfallah), Caliphate (calph), Banning of Caricatures (carc) of the Prophet (PBUH), Stopping Drone Strikes (drnst), Banning Gay Marriage in Muslim Countries (gaymrg), Availability of Halal Food in Spain (hlfd), Islamic Teaching in Spanish School (istch), Waging Armed Jihad (jihd), Liberating Kashmir (kash), Mosque Building (msqbl), Palestinian Right of Return (plror), Independent Palestinian State (plstat), Sharia (sharia), Expelling US Forces From Muslim lands (usmlt).

Fig S2A shows how many of our respondents would not forsake their position for monetary compensation. Fig S2B depicts how many of them would not give up their position if the majority opinion would change (*social immunity*). Opposition to caricatures, drone strikes, marriage equality were (almost) universally regarded as sacred, while availability of halal food and Islamic teaching as well as building Mosques without restrictions were not regarded as sacred by most respondents.

**Figures S2.** **Assessment of value sacredness and social immunity in the pre-selection sample**. **(A)** Percentage of respondents who consider the following demands sacred: Belief in Allah (blfallah), Caliphate (calph), Banning of Caricatures (carc) of the Prophet (PBUH), Stopping Drone Strikes (drnst), Banning Gay Marriage in Muslim Countries (gaymrg), Availability of Halal Food in Spain (hlfd), Islamic Teaching in Spanish School (istch), Waging Armed Jihad (jihd), Liberating Kashmir (kash), Mosque Building (msqbl), Palestinian Right of Return (plror), Independent Palestinian State (plstat), Sharia (sharia) and Expelling US Forces From Muslim lands (usmlt). **(B)** Percentage of respondents who would not give up their position if the majority opinion would change (social immunity).

Respondents also indicated which actions they would undertake on behalf of the issues: "Persuade people one-on-one," "Protest or apply public pressure for governments to find a diplomatic solution," "Protest or apply public pressure for governments to find a military solution (if issue in other country)/violently protest (if issue in same country)," "Support in spirit or financially a non-state militant group," "Join a non-state militant group," and "Carry out militant actions to fight and die on my own, even if I had no group or support network." On average, respondents were willing to make *M* = 2.6 (*SD* = 1.58, Cronbach's alphas .61-.77) sacrifices across all demands.

## S1. 3. Selection of fMRI participants

As the goal of fieldwork was to identify supporters of militant global jihadism for the fMRI study, we applied a series of increasingly strict criteria related to degree of individual radicalization: agreement with militant jihadi ideology, (tacit) approval of violence against civilians in the name of jihadism, actual willingness to advocate or engage in violence for jihadism.

To assess support for jihadi ideology, we focused on a main tenet of salafi jihadism (2), namely defense of the Muslim community (*ummah*) from external threats by means of violence. We assessed this with the statement "Armed jihad should be waged against those who seek to harm Muslims," which *n* = 121 respondents agreed with.

To gauge tacit approval for violence against civilians we examined respondents' opinion on Lashkar-e-Taiba, a Pakistan-based jihadi organization, familiar to all respondents. Lashkar-e-Taiba (lit. *Army of the Righteous*) is the largest and most active jihadi militant organizations in South Asia, headquartered in in Pakistan with several camps in Pakistan-administered Kashmir. It seeks to establish an Islamic state in South Asia. Among the most prominent attacks attributed to the organization the 2001 attack on the Indian parliament and the 2008 Mumbai attack. Among respondents, 49 had a somewhat or very favorable view of the organization, signifying tacit approval of its use of violence against civilians.

In addition to agreement with jihadism and approval of violence against civilians, we only considered respondents for the fMRI study who also expressed willingness to personally advocate or use violence to defend jihad (see above for complete list): to engage in violent protest (*n* = 110), to join a non-state militant group (*n* = 27), to fight and die on their own (*n* = 51).

**Figures S3.** **Opinions and attitudes of the selected vs. non-selected sample.** **(A)** Opinion on Lashkar-e Taiba across the pre-selection sample. Comparison between the selected and non-selected sample with respect to: **(B)** likelihood of being a devoted actor across all demands, **(C)** willingness to make costly sacrifices across demands and **(D)** willingness to make costly sacrifices for jihad.

From those *n* = 45 respondents who met all three criteria, N = 30 agreed to participate in the fMRI study. In the following, we provide comparisons between the respondents who participated in the fMRI study and those who did not (including those who met the criteria but did not agree to participate).

## S1. 4. Comparing fMRI participants to other respondents (including those who met the criteria but declined to participate).

Overall, fMRI participants did not differ in total number of issues they considered sacred (*M* = 7.88, *SD* = 1.3) from those who did not participate (*M* = 7.36, *SD* = 1.6), *t*(134) = 1.48, *p* = .141. Conversely, fMRI participants did not consider *M* = 6.2 (*SD* = 1.6) of the 14 given issues sacred, so providing a set of comparison values to choose from for the fMRI study design (within-subject comparison of processing of sacred vs. non-sacred values).

We ran a mixed-effect logistic model of being devoted (i.e., considering issues sacred AND being fused to a kin-like group of friends), which is comparable with an ordinary logistic regression but adjusted for dependency of the responses because of repeated measurement (sacredness and sacrifice measures) and the random error (noise) introduced by the specific set of issues probed (random effects). A dummy variable that encoded participation in the fMRI study (fixed effect) showed fMRI participants more likely to be devoted across all demands, Chi-Sq(*df* = 1) = 28.2, p < .001 (comparison with a null model excluding the dummy variable, Fig S3B).

A random intercept model of sacrifices also showed that, across all issues, fMRI participants were willing to make more sacrifices, Chi-Sq(*df* = 1) = 51.6, p < .001 (Fig S3C). For Jihad specifically, selected respondents were willing to make more costly sacrifices, *t*(134) = 4.00, p < .001 (to be expected as a result of our selection criteria) (Fig S3D).

Finally, a MANOVA with the Big Five items as dependent variables and participation in the fMRI study as the independent variable was not reliable, revealing that fMRI participants did not differ in personality from other respondents. Similarly, there were no reliable differences between the fMRI participants and other respondent in demographics or religiosity (Table S1).

**Table S1. Demographics and Religiosity of fMRI participants and Other Respondents**

|  |  | fMRI |  | Others |
| --- | --- | --- | --- | --- |
| Variable | Mean | SD | Mean | SD |
| Age | 29.67 | 5.45 | 31.49 | 7.73 |
| Education | 3.27 | 0.94 | 3.51 | 1.33 |
| Income | 1.30 | 0.53 | 1.25 | 0.59 |
| Frequency of Mosque Attendance | 3.47 | 0.82 | 3.22 | 0.76 |
| Frequency of Private Prayer | 3.37 | 1.38 | 3.05 | 1.88 |
| Importance of Religion | 6.30 | 1.15 | 6.36 | 1.19 |

## S1. 5. Measures of the pre-selection field survey

The pre-selection field survey consisted of a number of measures, which were adjusted if necessary during fieldwork to ensure that they were culturally appropriate and easily. We assessed sacredness of 14 issues (adjusted from previous measure, as described above), social immunity (refusal to give up value when the majority of peers changes opinion)(*14*), willingness to make costly sacrifices for each given issue, identity fusion with a number of groups (*3*), intergroup formidability, brief Big Five personality inventory (to compare the fMRI participants to others), a radical attitudes inventory, and demographics.

### *Sacred Values*

Based on fieldwork the following 14 issues were identified as relevant to the Pakistani community in Barcelona: ”India should have no right to ownership of Kashmir,” "Marriage between homosexual partners should be illegal in Muslim countries,” "All US and other Western military forces should be expelled from all Muslim lands-,” "There should be an immediate stop to all U.S. drone strikes in the northwestern frontier provinces,” "Palestinians have a right to return to their homes in Israel,” "There should be no Israel and only one Palestinian state from river to sea,” ”The Spanish government should make halal food available in public centers (schools, hospitals, shelters, prisons),” "The Spanish government should offer Islamic teachings to Muslim children in public schools,” "The Spanish government should allow the unrestricted building of mosques,” "The strictest form of Sharia should be applied in all Muslim lands,” "The boundaries of current Muslim countries should be dismantled and replaced with a single Caliphate,” "Armed jihad should be waged against the enemies of Muslims,” "Everyone must profess that Allah is the one and only true God and Mohamed (PBUH) is his messenger,” "Prophet Mohammed (PBUH) must never be caricatured."

Sacredness was assessed in a 2-step procedure. First, respondents indicated agreement or disagreement with a given statement. Then, respondents were presented with specific, realistic examples of trading off the given value for material gain. For example, if a participant agreed that "India should have no rights to ownership of Kashmir" then the participant would be presented with the following example (translated from Urdu):

"If instead of allowing Kashmir to belong to Pakistan or become independent the Indian government gave large amounts of tax refunds, local aid, heavy economic investment (for example, more jobs), and other monetary compensation to all Kashmiri families so they could live a higher material quality of life, or any other purely financial incentives, to stay part of India, would you find it acceptable?"

Responses involved choosing between "Yes," "Maybe," or "No." Respondents who rejected the trade-off (i.e., chose "No") were categorized as holding the given issue as a sacred value.

### *Social Immunity*

Social immunity was assessed by probing if the respondent would agree to compromise on the given issue when a majority of people agreed to this. For instance, on the Kashmir issue, they were asked: "What if the majority of Kashmiris wanted to stay part of India. Would that make it alright for India to have ownership of Kashmir?” Respondents chose between "Yes," "Maybe," and "No." Respondents who chose "No" were categorized as being immune to social pressure on the given issue.

### *Costly Sacrifices*

For each of the 14 issues, respondents were asked: “Please select all the actions that you would be willing to take to defend your position on this issue.” They could choose from: “1 - Nothing at all,” “2 - Persuade people one-on-one,” “3 - Protest or apply public pressure for governments to find a diplomatic solution,” “4 - Protest or apply public pressure for governments to find a military solution (if issue in other country)/violently protest (if issue in same country),” “5 - Support in spirit or financially a non-state militant group,” “6 - Join a non-state militant group,” “7 - Carry out militant actions to fight and die on my own, even if I had no group or support network.”

At the end of the Sacred Values and their Costly Sacrifices section, participants ranked the values. Ranking all 14 values on one list proved difficult for our population, so we reduced the number of values to be ranked to those for which the respondent refused trade-offs or showed social immunity.

### *Identity Fusion*

We assessed Identity Fusion (pictorial measure, Fig S4, upper image) with the following groups that were relevant in the context of the given 14 issues: Islam, Muslim Ummah, Pakistan, Caliphate (borderless or federation), India, Israel, USA, Spain, Close friends outside of immediate family. After participants indicated their degree of identity fusion with each group, they ranked the groups based on order of personal importance.

**Fig S4.** **Complementary measures.** Upper image: Identity fusion scale including two circles representing the self (small circle) and the group (larger circle) with increasing degrees of overlapping representing the participant’s relationship with the group. Bottom image: Physical and Spiritual Intergroup Formidability measure by means of male body representations with increasing size and strength.

### *Intergroup Formidability*

Intergroup Formidability was assessed with a pictorial measure depicting a male body with increasing strength and size (*12*) (Fig S4, bottom image). The formidability of the USA, Israel, India, and Spain were assessed vis-à-vis with Pakistan. Participants were first asked to evaluate the physical formidability and then the spiritual formidability of the given groups (spiritual and physical formidability were assessed with exactly the same body images but within different verbal frames).

### *Radical Attitude Inventory*

Additional exploratory questions concerned political awareness, perceptions of ideal Muslim countries, threat perception, in-group exceptionalism, support for strict Sharia law, support jihadist groups, and other attitudes linked to radicalization. Most of these measures are relevant for other projects but not here. Most importantly, we embedded Lashkar-e-Taiba as one of the groups we assessed favorability ratings for.

### *Brief Big Five Personality Inventory*

A validated 10-item Big Five personality inventory allowed for comparisons between fMRI respondents and others.

### *Demographics and other miscellaneous measures*

Demographic questions concerned age, marital status, education, income, prayer frequency, political leanings. Additional questions probed political awareness, perceptions of ideal Muslim countries, and other issues.

## S1. 6. Multivariate pattern analysis

To corroborate the univariate results, a multivariate pattern analysis (MVPA) method known as searchlight decoding analysis was implemented by means of the The Decoding Toolbox (TDT) in SPM12. This analysis was used to identify brain clusters containing relevant information to distinguish between i) willingness to fight and die for sacred and non-sacred values values, ii) peers-lower feedback and peers-agree feedback on willingness to fight and die for sacred values, iii) peers-lower feedback on willingness to fight and die for sacred and non-sacred values. A regression analysis was also conducted with self-reported moral outrage as predictor of informative neural regions in the classification between i) peers-lower feedback and peers-agree feedback on willingness to fight and die for sacred values, and ii) peers-lower feedback on willingness to fight and die for sacred and non-sacred values.

Unlike univariate methods predicting one single dependent variable (the BOLD activation signal) for every voxel in the brain, MVPA methods simultaneously predict multiple dependent variables (BOLD activity in multiple voxels) as a function of one or more independent variables. Conjointly analyzing activity in multiple voxels allows us to account for their covariance, thus increasing odds of finding statistical dependency. The decoding searchlight analysis can distinguish two highly similar brain states, making use of a support vector machine learning algorithm trained to classify distinct patterns of activity in a previously defined training set containing both brain states. The trained classifier is then tested to classify a different sample of unlabeled brain states. As a result, an above-chance classification accuracy rate ranging between 50% (classification at chance level) and 100% (all classifications correct) is extracted for each voxel in the brain. Such accuracy rate is a measure of how distinguishable two brain states are based on information contained in each brain location.

Here, we ran the same pre-preprocessing steps as in the univariate analysis with the exception of the smoothing step, since smoothed images are inconvenient for the MVPA analysis (8). We performed a searchlight analysis on each subject with a searchlight radius of 12mm. In order to increase statistical power, a leave-one-out cross-validation was conducted; the classification process was systematically repeated for four rounds taking 75% of the trials as the training set and 25% as a testing set in each round, so that the each time the testing set was composed of untested trials only. Finally, we extracted group-level effects by running a one-sample t-test on the classification accuracy maps of all participants and thresholded the results at p <.05 FWE at cluster-level (single voxel p <.001).

# S2. MVPA results

In addition to the univariate analysis results (see Table S2), the MVPA analysis identified clusters within the dlPFC, the dmPFC, and the parietal cortex as significant nodes distinguishing willingness to fight and die decisions over sacred versus non-sacred values (T = 5.2, p < .05 FWE; see Table S3), corroborating between-condition differences in areas involved in reasoning and cognitive control (*20*).Moreover, the MVPA analysis of the false consensus feedback paradigm revealed differential activity in the left parietal operculum between the conflicting and non-conflicting feedback condition for sacred values (T = 3.12, p < .05 FWEc, single voxel p < .001). As reported in the main text, differential activity in the same area was successfully predicted by self-reported moral outrage at conflicting feedback for sacred values in the contrast between conflicting feedback for sacred and non-sacred values (T = 3.12, p < .05 FWEc, single voxel p <. 001, see Table S3).

Table S2. Results of the Rating 1 univariate analysis. These include neural clusters surviving a p < .05 FWE correction across the whole-brain for the contrasts: sacred values > fixation cross, non-sacred values > fixation cross and non-sacred values > sacred values.

| Univariate analysis |  | | Peak MNI coordinates | | |
| --- | --- | --- | --- | --- | --- |
|  | extent | t-value | x | y | z |
| Sacred values < non-Sacred values | | | | | |
| L dorsolateral prefrontal cortex | 7416 | 7,72 | -30 | 14 | 44 |
| L inferior frontal gyrus (p. Triangularis) | 7416 | 6,46 | -52 | 18 | 36 |
| R dorsolateral prefrontal cortex | 2685 | 5,16 | 36 | 32 | 48 |
| R inferior frontal gyrus (p. Opercularis) | 2685 | 4,67 | 58 | 14 | 10 |
| L superior parietal lobule | 2682 | 6,76 | -26 | -70 | 60 |
| L inferior parietal lobule | 2682 | 5,25 | -40 | -54 | 60 |
| L middle occipital gyrus | 2682 | 5,05 | -34 | -68 | 38 |
| L fusiform gyrus | 4106 | 6,06 | -40 | -74 | -8 |
| R cerebelum (Crus 1) | 4106 | 5,71 | 36 | -74 | -24 |
| L cerebelum (Crus 1) | 4106 | 5,42 | -16 | -80 | -22 |
| R angular gyrus | 2691 | 5,61 | 38 | -74 | 48 |
| R inferior parietal lobule | 2691 | 5,33 | 44 | -44 | 60 |
| R superior parietal lobule | 2691 | 5,21 | 26 | -58 | 54 |
| R caudate nucleus | 397 | 5,50 | 14 | 4 | 16 |
| Sacred values > baseline | | | | | |
| R superior temporal gyrus | 8811 | 9,18 | 62 | -42 | 26 |
| R middle temporal gyrus | 8811 | 8,79 | 56 | -60 | 16 |
| R linual gyrus | 8811 | 8,15 | 28 | -54 | -2 |
| L middle temporal gyrus | 584 | 6,20 | -40 | -68 | 22 |
| R middle orbital gyrus | 928 | 5,68 | 2 | 40 | 4 |
| L middle cingulate cortex | 454 | 4,95 | -8 | -44 | 50 |
| Willingness to fight and die for sacred values: positive correlation (small volume correction) | | | | | |
| L middle orbital gyrus | 13 | 4,05 | 0 | 54 | -4 |
| Change in judgment (masked with non-sacred value>sacred value contrast thresholded at p<0.001) | | | | | |
| R dorsolateral prefrontal cortex | 319 | 5,13 | 38 | 8 | 56 |

**Table S3.** **Results of the Rating 1 and Feedback multivariate analysis.** These include clusters with differential activity surviving a p<.05 FWE correction across the whole-brain for the classifications: sacred values vs non-sacred values, peers-lower feedback (sacred values) vs peers-agree feedback (sacred values), and activity differences predicted by moral outrage in the classification peers-lower feedback (sacred values) vs peers-lower feedback (non-sacred values).

| Multivariate analysis |  | | Peak MNI coordinates | | |
| --- | --- | --- | --- | --- | --- |
|  | extent | t-value | x | y | z |
| Sacred values vs. non-sacred values | | | | | |
| R supramarginal | 289 | 6,61 | 50 | -38 | 38 |
| L inferior frontal triangularis | 591 | 6,42 | -48 | 24 | 12 |
| L parietal superior/parietal inferior | 626 | 6,40 | -20 | -70 | 54 |
| L cerebellum | 364 | 6,33 | -34 | -66 | -26 |
| R precuneus | 179 | 6,23 | 10 | -64 | 66 |
| R dorsolateral prefrontal | 189 | 6,19 | 24 | 34 | 44 |
| L dorsolateral prefrontal | 180 | 6,17 | -36 | 12 | 56 |
| L supplementary motor area | 60 | 6,04 | -6 | 14 | 62 |
| Peers-lower vs. peers-agree feedback (sacred values) | | | | | |
| L parietal operculum | 101 | 4,16 | -54 | -16 | 22 |
| R cuneus | 106 | 4,13 | 26 | -60 | 22 |
| Moral outrage predicting Peers-lower feedback for sacred values vs. for non-sacred values | | | | | |
| L supramarginal/parietal operculum | 59 | 4, 35 | -56 | -22 | 18 |

**Supporting Figures**

**Fig S5.** **Correlation matrix of post-manipulation emotions in the sacred value condition for peers-agree and peers-lower community feedback.** Outrage and shame showed floor effects in the peers-agree condition and were thus not included in the correlation analysis. The color bar represents Pearson’s coefficients r. Several observations could be made: a) High consistency among negative (outrage, shame) and among positive emotions (joy, compassion, pride) in all feedback conditions (red blocks). b) Outrage and shame towards the in-group after peers-lower feedback correlated with positive emotions after peers-agree feedback (orange and yellow blocks) c) Shame and pride after peers-lower feedback were negatively correlated (in blue). * p < 0.05, ** p < 0.001, n.s. = non-significant correlation.

**Fig S6. Follow-up survey results. (A)** Higher reported anger at having to defend sacred values (*M* = 6.27, *SD* = 0.64) than non-sacred values (*M* = 1.79, *SD* = 0.65) (*t*(17) = 24.79, *p* < .001). **(B)** Higher reported joy at having to defend sacred values (*M* = 6.54, *SD* = 0.43) than non-sacred values (*M* = 3.30, *SD* = 1.04) (*t*(17) = 13.265, *p* < .001). **(C)** Higher reported excitement at having to defend sacred values (*M* = 6.52, *SD* = 0.43) than non-sacred values (*M* = 3.28, *SD* = 1.01) (*t*(17) = 13.47, *p* < .001). **(D)** Higher reported emotional intensity at having to defend sacred values (*M* = 6.44, *SD* = 0.48) than non-sacred values (*M* = 2.79, *SD* = 0.82) (*t*(17) = 18.15, *p* < .001). **(E)** Higher reported familiarity with issues that were sacred values (*M* = 3.67, *SD* = 0.21) than non-sacred values (*M* = 2.62, *SD* = 0.25) (*t*(17) = 28.13, *p* < .001). **(F)** Higher reported frequency of thinking about sacred values (*M* = 3.53, *SD* = 0.34) than non-sacred values (*M* = 2.09, *SD* = 0.33) (*t*(17) = 20.91, *p* < .001). **(G)** Higher reported mental energy thinking about sacred values (*M* = 3.51, *SD* = 0.20) than non-sacred values (*M* = 2.16, *SD* = 0.25) (*t*(17) = 18.01, *p* < .001)**.(H)** Higher reported saliency with issues that were sacred values (*M* = 3.54, *SD* = 0.20) than non-sacred values (*M* = 2.13, *SD* = 0.26) (*t*(17) = 23.92, *p* < .001).

**Fig S7.** **Results of the two strategies aimed to control for the confounding effects of emotional intensity, familiarity and salience on the sacred value < non-sacred value contrast.** **(A)** Results of the non-sacred value > sacred value contrast survived in the 3 follow-up GLMs (N = 18) that included, respectively, parametric regressors for emotional intensity (average of anger, excitement and joy ratings) and reaction times (upper image), parametric regressors for familiarity and reaction times (middle image), and parametric regressors for salience and reaction times (bottom image). **(B-C)** Results of the sacred value < non-sacred value contrast using the original GLM (N = 29) survived after masking out results with six masks comprising brain regions that positively and negatively correlate with **(B)** emotional intensity, familiarity and saliency scores obtained from the follow-up GLMs using a threshold of p <.01, and **(C)** “emotion”, “familiarity” and “salience” obtained from the Neurosynth based on a meta-analysis of 11406 studies.

**Fig S8. Binary masks used to exclude neural activity correlated with reaction time (in cyan), emotions (in yellow), familiarity (in blue) and salience (in red).** Masks were calculated based on the neural correlates of reaction times in the original GLM **(A)**, emotional intensity, salience and familiarity revealed by the follow-up GLMs **(B)** and defined using Neurosynth **(C)**, a database of 11406 studies that provides probability maps that reflect the likelihood that the terms “emotion”, “familiarity” and “salience”, respectively, were used in a study based on the presence of activity in a given voxel (thresholded at Z = 5).
